# Expression and localization of estrogenic type 12 17β-hydroxysteroid dehydrogenase in the cynomolgus monkey

**DOI:** 10.1186/1471-2091-8-2

**Published:** 2007-02-05

**Authors:** Hong Liu, ShuFang Zheng, Véronique Bellemare, Georges Pelletier, Fernand Labrie, Van Luu-The

**Affiliations:** 1 Oncology and Molecular Endocrinology Laboratory, Laval University Hospital Research Center (CRCHUL) and Laval University, Quebec, GlV 4G2, Canada

## Abstract

**Background:**

We have recently discovered that human type 12 17β-HSD (h17β-HSD12), a homolog of type 3 17β-HSD, is a new estrogen-specific 17β-hydroxysteroid dehydrogenase involved in the production of estradiol (E2). To further characterize this estradiol-producing enzyme, we have isolated the corresponding cDNA in the cynomolgus monkey (*Macaca fascicularis*), characterized its enzymatic activities and performed cellular localization using *in situ *hybridization.

**Results:**

Using HEK-293 cells stably expressing *Macaca fascicularis *type 12 17β-HSD (*mf*17β-HSD12), we have found that the *mf*17β-HSD12 catalyzes efficiently and selectively the transformation of El into E2, in analogy with the h17β-HSD12. We have also quantified the *mf*17β-HSD12 mRNA expression levels in a series of *Macaca fascicularis *tissues using Quantitative RealTime PCR. The *Macaca fascicularis *17β-HSD12 mRNA is widely expressed with the highest levels tissues found in the cerebellum, spleen and adrenal with moderate level observed in all the other examined, namely the testis, ovary, cerebral cortex, liver, heart, prostate, mammary gland, myometrium, endometrium, skin, muscle and pancreas. To gain knowledge about the cellular localization of the *mf*17β-HSD12 mRNA expression, we performed *in situ *hybridization using a ^35^S-labeled cRNA probe. Strong labeling was observed in epithelial cells and stromal cells of the mammary gland. In the uterus, the labeling is detected in epithelial cells and stromal cells of the endometrium.

**Conclusion:**

These results strongly suggest that the *Macaca fascicularis *17β-HSD12 is an essential partner of aromatase in the biosynthesis of estradiol (E2). It strongly suggests that in the estradiol biosynthesis pathway, the step of 17-ketoreduction comes after the step of the aromatization (the aromatization of 4-androstendione to estrone followed by the conversion of estrone into estradiol by estrogen specific l7β-HSDs) which is in contrast with the hypothesis suggesting that 4-androstenedione is converted to testosterone followed by the aromatization of testosterone.

## Background

Seventeen β-hydroxysteroid dehydrogenases (17β-HSDs) are crucial enzymes involved in the formation of active sex steroids by the transformation of a keto into a hydroxyl-group at position 17. The best known 17β-HSD is type 3 17β-HSD (17β-HSD3) that is expressed in the testis where it transforms androstenedione (4-dione) into testosterone (T). Its deficiency is the cause of the well known male pseudohermaphroditism [4,5]. This enzyme is inactive for C18-steroids. An additional enzyme able to catalyze the transformation of 4-dione into T is type 5 17β-HSD [6,7]. Since 17β-HSD3 is not expressed in the ovary while 17β-HSD5 is present [8,9], and women deficient in 17β-HSD3 are asymptomatic [10], it is likely that 17β-HSD5 is the enzyme responsible for the formation of active androgens in the human ovary [11].

On the other hand, the most studied 17β-HSD is type 1 17β-HSD, probably because it is expressed abundantly in the placenta. In fact, 17β-HSD1 was the first 17β-HSD to be purified [12], cloned [13,14] and crystallized [15]. In intact cell in culture, the enzyme catalyzes almost exclusively the transformation of estrone (El) into estradiol (E2) [11,16]. The abundant co-expression of this estrogenic 17β-HSD as well as aromatase in the placenta suggests that the aromatization step precedes the 17β-HSDs step. This proposed mechanism is also in agreement with a higher affinity of aromatase for 4-dione than for testosterone. Other 17β-HSDs are known to be able to metabolize estrogens. Thus types 7 and 12 17β-HSDs catalyze the formation of E2, types 2, 4, and 8 17β-HSDs that preferred NAD^+ ^as cofactor are E2 inactivating enzymes [11].

Up to now, at least twelve isoforms of 17β-HSDs have been identified and some members of the 17β-HSDs family have been shown multifunctionality associated with cancer, metabolism diseases and neurodegenerative disorders, in addition to their roles in steroid metabolism [1-3]. Most of 17β-HSDs belong to the short-chain dehydrogenase reductase (SDR) superfamily except the type 5 17β-HSD, which belongs to the aldo-keto reductase (AKR) superfamily. A particular property of members of 17β-HSDs family is that they possess very different primary structures (an average of only approximately 20% amino acid identity) despite being highly specific for substrates having closely related structures. Additional regulation of 17β-HSDs activity is achieved by the specificity of tissue distribution of these 17β-HSDs, thus permitting each tissue to control intracellular steroid levels according to local needs. Such local intracellular formation of steroids in peripheral target tissues from the adrenal precursor dehydroepiandrosterone (DHEA) has been called intracrinology [17,18].

Recently, we have found that the h17β-HSD12, a homolog of type 3 17β-HSD, selectively catalyzes the formation of E2 [19]. To gain more knowledge about this potentially very important enzyme, we have isolated a corresponding enzyme in the cynomolgus monkey (*Macaca fascicularis*), and characterized its substrate specificity, mRNA tissue distribution and cellular localization.

## Results

### Sequence of Maraca fascicularis 17β-HSD12

**Figure 1:**
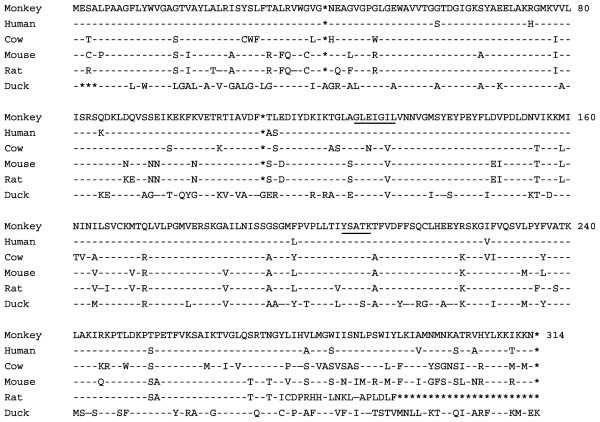
**Alignment of the amino acid sequence of 17β-HSD12 of *Macaca fascicularis *and other species**. The deduced amino acid sequence of *Macaca fascicularis *17β-HSD12 was aligned with the human, cow, mouse, rat and duck counterparts. The amino acid sequences are presented in the convention single letter code and numbered on the right. Dashes (-) and asterisks (*) represent identical and missing amino acid residues. The conserved sequences for co-factor binding and active sites are underlined.

We have isolated a coding sequence of *Macaca fascicularis *17β-HSD12 (GenBank accession number AB169576) using *Macaca fascicularis *liver mRNA and PCR amplification. As illustrated in Fig. 1, amino acid sequence alignment of 17β-HSD12 between *Macaca fascicularis *and other species shows that the *Macaca fascicularis *sequence possesses 95%, 82%, 81%, 78% and 66% identity with human, cow, mouse, rat, and duck, respectively. In addition, this enzyme contains the conserved signatures of SDR family members, namely the putative YXXXK active center and the modified GXXXGXL cofactor binding site.

### Substrate specificity of Macaca fascicularis 17β-HSD12

**Figure 2:**
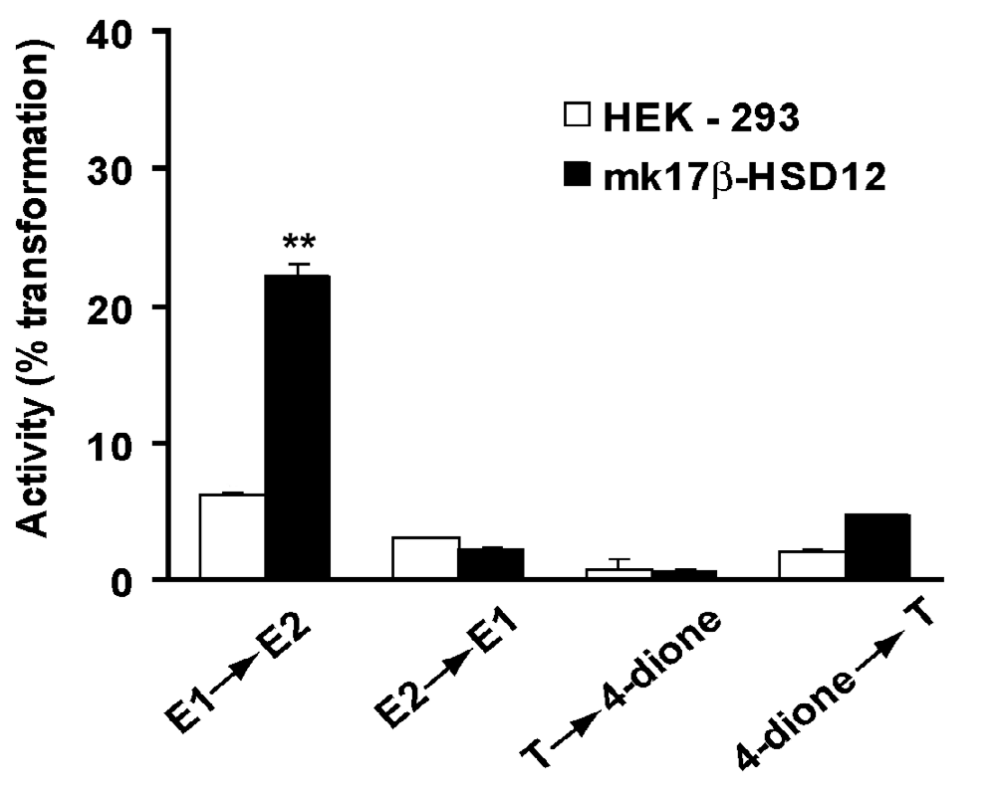
**Determination of the enzymatic activities of *Macaca fascicularis *17β-HSD12 in intact transfected 293 cells in culture**. Cells stably transfected with *mf*17β-HSD12 were seeded into 6-well plates at a density of 5 × 10^5 ^cells/well. 0.1 μM of [^14^C]-labeled El, E2, 4-dione, and T were added to freshly changed culture medium. Non transfected HEK-293 cells were used as controls. After 20 h of incubation, the media were collected and extracted. The data are expressed as a mean ± SEM of triplicate measurements. ** indicates significantly different from non transfected HEK-293 cells at p < 0.01.

**Figure 3:**
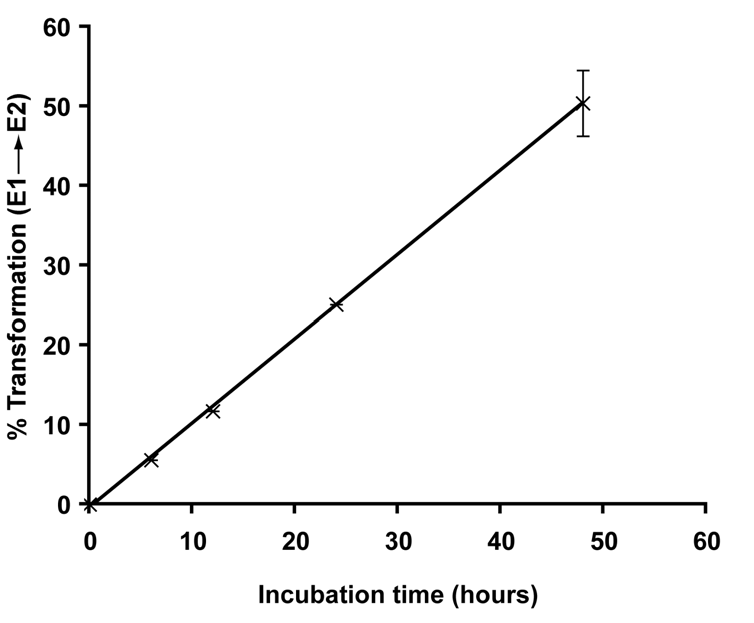
**El to E2 conversion rate *of Macaca fascicularis *17β-HSD12**. HEK-293 cells stably transfected with *mf*17β-HSD12 were incubated with 0.1 μM [^14^C]-labeled El. After incubation for 8, 12, 24 and 48 h, the media were collected and extracted. Non transfected HEK-293 cells were used as control. (---x---) represent values over control. The data are expressed as means of duplicate measurements.

We used the HEK-293 cells stably expressing *mf*17β-HSD12 to determine the substrate specificity of the enzyme in intact cells in culture without addition of exogenous cofactor. As shown in Fig. 2, in analogy with the h17β-HSD12, the *mf*17β-HSD12 catalyzes predominately the transformation of El into E2 while the transformation of 4-dione into T, E2 into El and T into 4-dione are not significant. Fig. 3 shows that the conversion of El into E2 increases proportionally with the incubation, thus indicating that our incubation conditions are appropriate and that time is a limiting step even after 50 h of incubation.

### Tissue distribution of Macaca fascicularis 17β-HSD12

**Figure 4:**
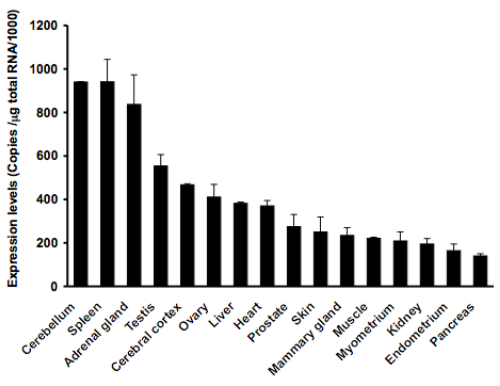
**mRNA expression levels of *Macaca fascicularis *17β-HSD12 measured by Q_RTPCR**. mRNA expression levels were quantified in the adrenal gland, ovary, mammary gland, endometrium, myometrium, cerebral cortex, cerebellum, liver, pancreas, heart, testis, prostate, kidney, skin, muscle and spleen by Quantitative RTPCR (Q_RTPCR). The reaction was performed using the amount of cDNA corresponding to 30 ng of initial total RNA following the manufacturer's protocol. All sample were run in duplicates and quantification of each target gene expression was done two or three times. Results are expressed as mean ± SEM.

Using quantitative RealTime PCR, we examined the expressions levesl and tissue distribution of 17β-HSD12 mRNA in 16 *Macaca fascicularis *tissues, namely the adrenal gland, ovary, mammary gland, endometrium, myometrium, cerebral cortex, cerebellum, liver, pancreas, heart, testis, prostate, kidney, skin, muscle and spleen. As illustrated in Fig. 4, the *mf*17β-HSD12 is ubiquitously expressed with the highest level in the spleen, adrenal gland and cerebellum, and moderate levels all the other tissues examined. *Macaca fascicularis *17β-HSD12, which is highly and widely expressed in gonadal as well as extragonadal tissues is most probably a crucial enzyme involved in the biosynthesis of estradiol.

### Cell-specific distribution of Macaca fascicularis 17β-HSD12

**Figure 5:**
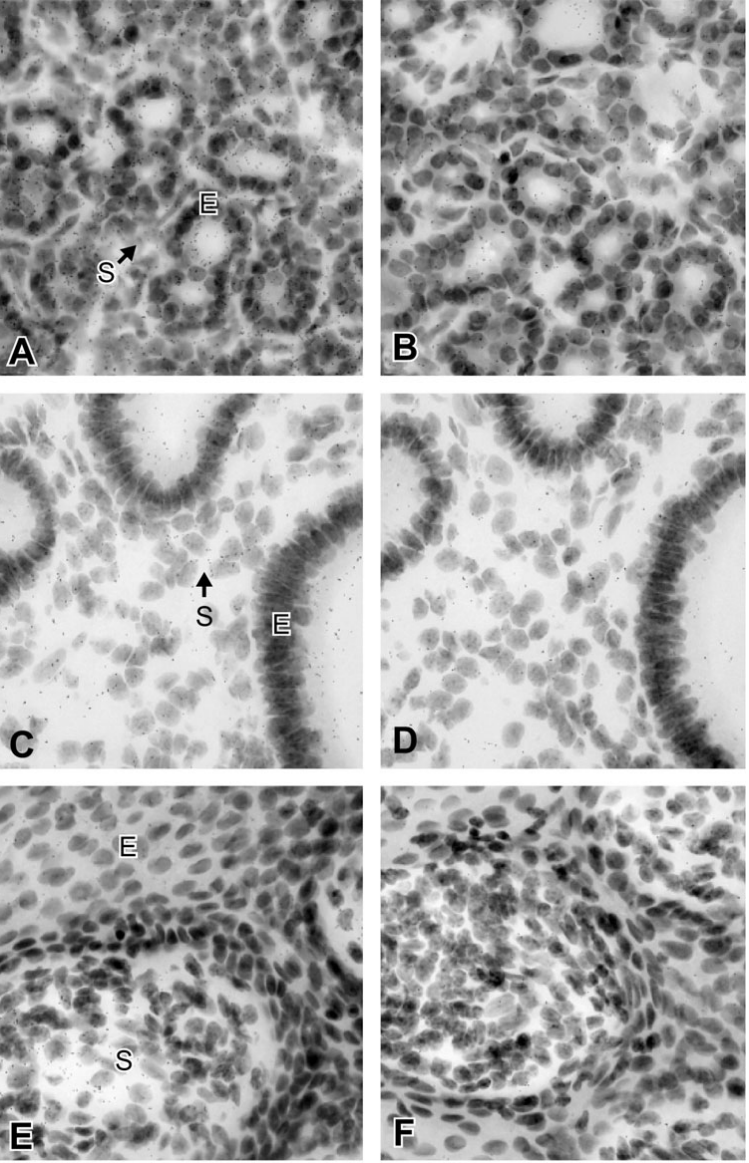
**Cell-type specific expression of 17β-HSD12 mRNA in the *Macaca fascicularis *mammary gland (A-B) and uterus (C-F) revealed by *in situ *hybridization**. (A) Section through the mammary gland of the female *Macaca fascicularis*. Labeling can be observed in the epithelial cells of alveoli (E) as well as in the stromal cells (S). (B) Control section hybridized with the sense probe. Only a diffuse background is observed. Exposure, 36 days, × 600. (C) Section through the uterus *Macaca fascicularis*. Labeling can be detected in both epithelial cells (E) and stromal cells (S) of the endometrium. (E) Section through the uterine cervix, labeling can be seen in squamous epithelial cells (E) and stromal cells (S). Control section hybridized with the sense probe (D, F). Diffuse background can be observed (D). Only a few disperse silver grain are present in the uterine cervix (F). Exposure, 27 days, × 600.

In order to obtain information about the cellular localization of 17β-HSD12 in estrogen-sensitive tissues, we performed *in situ *hybridization on the adult female *Macaca fascicularis *mammary gland and uterus using a ^35^S-labeled probe. The labelling was detected on both the epithelial cells of the alveoli and the stromal cells of the mammary gland (Fig. 5A). In the uterus, the hybridization signal was seen in the epithelial cells and in the stromal cells of the endometrium (Fig. 5C). In the uterine cervix, 17β-HSD12 mRNA was observed in the squamous epithelium and stromal cells (Fig. 5E). When the radiolabeled sense probes were used for hybridization in consecutive sections, only weak and diffuse labelling corresponding to background could be detected in the mammary gland, uterus and uterine cervix (Fig. 5B, D and 5F).

## Discussion

In this report, we have shown that *Macaca fascicularis *17β-HSD12 catalyzes selectively the transformation of El into E2, similar to that has been found for the corresponding human enzyme. The present data strongly suggest that 17β-HSD12 is most likely the key enzyme controlling the local conversion of El (low estrogenic activity) into E2 (the most potent natural estrogen). It is noteworthy that El could come from both sources, the local transformation of the precursor 4-dione by aromatase or from the circulation under the form of El and El-S [20]. It is well recognized that most of peripheral cells possess all the necessary enzymatic machinery to transform the adrenal androgen precursors into E2 [17,18]. Cells that possess steroid sulfatase could use the sulfated precursor DHEA-S and El-S to produce E2.

The mRNA tissue distribution analysis performed with quantitative RealTime PCR shows that the enzyme is distributed ubiquitously, thus suggesting its important role in the production of estradiol in a large number and possibly all peripheral target tissues. In contrast 17β-HSD1 is more selectively expressed in the placenta [21] and in granulosa cells of the ovary [22,23]. The highest mRNA expression levels have been found in the cerebellum and spleen suggesting that this enzyme could play an important role in these tissues. However, in the tissues where a lower mRNA expression levels is observed, it could not mean that the role exerted by the enzyme is not important, but it reflects essentially the relatively fewer amount of cells expressing this enzyme compare to the total cells in the tissue.

Cloning of the cDNA of *mf*17β-HSD12 has allowed us to study the cellular localization of the enzyme in the mammary gland and uterus. In the mammary gland, 17β-HSD12 mRNA expression was detected in both epithelial and stromal cells. This is in agreement with the action of estrogens in the development and proliferation of alveoli and lactogenesis in the mammary gland. In the uterus, the enzyme was found in the both epithelial cells and stromal cells of the endometrium. These results strongly suggest that 17β-HSD12 is directly involved in the local synthesis of E2 in the estrogen target tissues.

Although twelve types of 17β-HSDs have been identified based on their specificity to catalyze the interconversion of the 17β-ketosteroids and 17β-hydroxysteroids, and named according to the chronological order of their characterization, the true physiological role of many of these enzymes remains to be determined. For example, the phenotype of Hsdl7b2 knockout mice is not well explained by the known activities of 17β-HSD2 on sex steroids [24]. On the other hand, 17β-HSD4, which was originally identified as the estradiol-inactiving dehydrogenasse from porcine endometrium [25,26], mainly participates in the β-oxidation of fatty acids [27]. Accordingly, mutations of the 17β-HSD4 gene lead to severe developmental defects resembling Zellweger syndrome [28], a rare hereditary disorder affecting infants, and usually results in death associated with unusual problems in prenatal development, an enlarged liver, high levels of iron and copper in the blood, and vision disturbances. Type 6 17β-HSD, an enzyme observed in the rat, most probably corresponds to type 9 17β-HSD in the mouse that catalyses the transformation of 5α-androstane-3α, 17β-diol (3α-diol) into androsterone (ADT). The corresponding human ortholog is not yet identified. On the other hand, type 7 17β-HSD is presently best characterized as a 3β-reductase involved in cholesterol metabolism [29], and the role of type 8 17β-HSD as an estradiol inactivating enzyme [11,30,31] is still not well defined. Types 10 and 11 l7β-HSDs metabolize 5α-reduced steroids but their involvement in the metabolism of active steroids is unclear. Type 12 17β-HSD was identified as 3-ketoacyl-CoA reductase, involved in long chain fatty acid elongation with a very weak activity [32]. Recently, we have found that the H17β-HSD12 catalyzes selectively the formation of E2 [19].

Overall, among the twelve enzymes identified as l7β-HSDs, only four are most likely working as l7β-HSDs synthesizing active steroids: the types 3 and 5 l7β-HSDs are involved in the transformation of C19-steroids (4-dione into T) while types 1 and 12 are involved in the conversion of El into E2. The selective activity of type 12 17β-HSD for the transformation of El into E2 in the *Macaca fascicularis *(this report) as well as the high mRNA expression levels of this enzyme in estrogen-sensitive tissues, including the mammary gland and uterus, strongly suggest that this enzyme is a crucial partner of aromatase in the biosynthesis of estradiol. Indeed, the presence of estrogen specific 17β-HSDs (types 1, 7 and 12 l7β-HSDs) catalyzing the transformation of El to E2 and the higher affinity of aromatase for 4-dione than for T are strongly in favor of the pathway in which 4-dione is converted into El by aromatase followed by the transformation of El into E2 by estrogen specific l7β-HSDs. This pathway is in contrast with a generally believed pathway in which 4-dione is transformed to T by l7β-HSDs followed by the aromatization of T into E2. It strongly suggests that in the estradiol biosynthesis pathway, the step of 17-ketoreduction comes after the step of the aromatization (the aromatization of 4-androstendione to estrone followed by the conversion of estrone into estradiol by estrogen specific l7β-HSDs). The higher affinity of aromatase for 4-androstenedione than for testosterone seems to agree with the present data.

## Conclusion

These results strongly suggest that the *Macaca fascicularis *17β-HSD12 is an essential partner of aromatase in the biosynthesis of estradiol (E2). It strongly suggests that in the estradiol biosynthesis pathway, the step of 17-ketoreduction comes after the step of the aromatization (the aromatization of 4-androstendione to estrone followed by the conversion of estrone into estradiol by estrogen specific l7β-HSDs) which is in contrast with the hypothesis suggesting that 4-androstenedione is converted to testosterone followed by the aromatization of testosterone.

## Methods

### Experimental animals

The maintenance and handling of experimental animals followed the National Institute of Health Guidelines for the use and care of animals and was done under approval and supervision of the Comité de Protection des Animaux du CHUQ (CPAC). The tissues used were harvested from euthanized normal female and male cynomolgus monkeys, frozen in liquid nitrogen and stored at -70°C until analysis.

### RNA isolation and cDNA synthesis

Total RNA was extracted from the *Macaca fascicularis *adrenal gland, ovary, mammary gland, endometrium, myometrium, cerebral cortex, cerebellum, liver, pancreas, heart, testis, prostate, kidney, skin, muscle and spleen by using Tri-Reagent RNA/DNA/Protein Isolation Reagent (Molecular Research Center Inc., Cincinnati, OH, USA). The extraction was performed according to the manufacturer's directions. Quantification was made by optical density at 260 nm. 5.0 μg of total RNA was converted to cDNA by incubation at 42°C for 1 h with 200 U of Superscript II RNase H-RT (Invitrogen, Burlington, Ont. Canada), 300 ng of oligo-dT18, 500 μM deoxynucleotide triphosphate, 10 mM dithiothreitol, and 34 U of porcine RNase inhibitor (Amersham Pharmacia) in a final volume of 50 μl. The resulting products were then treated with RNase A for 30 min at 37°C and purified thereafter with Qiaquick PCR purification kit (QIAGEN, Mississauga, Ontario).

### Isolation of Macaca fascicularis 17β-HSD12 cDNA

Since ortholog genes between the human and monkey possess generally more than 90–95% identity, we designed oligonucleotide primers based on the human sequence before the initiation codon (forward primer 5'-GTA-GTG-AGG-CCT-AGT-GGA-AAG-CCA-TG-3') and after stop codon (reverse primer 5'-CAA-GTT-ACA-ATG-CAG-TTA-TCA-TGC-3'). The fragment containing the entire *mf *17β-HSD12 open reading frame was obtained by reverse transcriptase PCR (RT-PCR) amplification from *Macaca fascucularis *liver mRNA. The resulting PCR product was directly subcloned into a Zero Blunt TOPO vector (Invitrogen, Burlington, Ont. Canada). Plasmid DNA was prepared using the Qiagen Mega kit (Qiagen, Chatsworth, CA, USA) following the manufacturer's protocol. The integrity of the construct was verified by automated dideoxynucleotide DNA sequencing using the Big Dye Terminator v3.1 Cycle Sequencing (ABI Prism, applied biosystem, Forster city, CA). The cDNA fragment was further transferred into PCMVneo expression vector in order to prepare stable transfected HEK-293 cells for activity characterization.

### Enzymatic activity was determined in intact HEK-293 cells stably expressing Macaca fascicularis 17β-HSD12

Stable expression of *mf*17β-HSD12 in transformed human embryonic kidney cells (HEK-293) was performed as previously described [16]. Enzymatic activities were determined with intact cells stably expressing *mf*17β-HSD12 in culture. Briefly, cells were plated in 6-well plates to approximately 5 × 10^5^/well in MEM. 0.1 μM of the [^14^C]-labeled steroids (Dupont Inc., Mississauga, Ont. Canada) were added to freshly changed culture medium and incubated for 20 h. Mock transfection was done with the plasmid DNA of the pCMVneo vector. After incubation, the steroids were extracted twice with 2 ml of ether. The organic phases were then pooled and evaporated to dryness. The steroids were dissolved in 50 μL of dichloromethane, applied to Silica gel 60 thin layer chromatography (TLC) plates (Merck, Darmstadt, Germany), before separation by migration in the toluene/acetone (4:1) solvent system. Substrates and metabolites were identified by comparison with reference steroids, revealed by autoradiography and quantified using the Phospholmager System (Molecular Dynamics, Inc., Sunnyvale, CA).

### mRNA expression by Quantitative RealTime PCR (Q_RTPCR)

*Macaca fascicularis *17β-HSD12 was amplified using the gene-specific primers: 5'-CAG-GCT-TGG-CTG-GTC-TTG-AA-3' and 5'-CAC-CAT-GCC-AGG-CAG-TAC-CAA-3'. cDNA corresponding to 30 ng of the initial total RNA was used to perform fluorescent-based RealTime PCR quantification using the LightCycler RealTime PCR apparatus (Hoffman-La Roche Inc. Nutley, NJ) as described [33]. The conditions for the PCR reactions were: denaturation at 94°C for 15 sec, annealing at 50°C for 10 sec and elongation at 72°C for 35 sec. The data were normalized using the mRNA expression levels of the *Macaca fascicularis *housekeeping gene glucose-6-phosphate dehydrogenase (G6PDH) as internal standard. The mRNA expression levels are expressed as number of copies/μg total RNA using a standard curve of Cp versus logarithm of the quantity. The standard curve is established using known cDNA amounts of 0, 10^2^, 10^3^, 10^4^, 10^5 ^and 10^6 ^copies of cDNA of glucose-6-phosphate dehydrogenase (forward primer: 5'-GGC-TGG-AAC-CGC-ATC-ATT-GTG-GA-3' and reverse primer: 5'-GGC-GAT-GTT-GTC-CCG-GTT-CCA-GA-3') and a LightCycler 3.5 program provided by the manufacturer (Roche Inc., Nutley, NJ). All sample were run in duplicates and quantification of each target gene expression was done two or three times. Results are expressed as mean ± SEM.

### In situ hybridization

Recombinant plasmid pCRII-TOPO (Invitrogen, Burlington, Ont. Canada), containing a 331 bp *Macaca fascicularis *17β-HSD12 fragment located at position 1–330 bp downstream from the ATG start codon was obtained by amplification using polymerase chain reaction. *In situ *hybridization with the antisense and sense ^35^S-labeled cRNA probes was performed as previously described [34,35]. After hybridization, the sections were dehydrated and coated with liquid photographic emulsion (Kodak-NTB2; diluted 1:1 with water). After 27–36 days of exposure, the sections were processed and counterstained with haematoxylin.

### Statistics

Results are given as mean ± SEM of two or three experiments. Data were analyzed by student's *t*-test for two columns. The differences was considered significant if p < 0.05.

## Abbreviations

17β-HSD 17β-hydroxysteroid dehydrogenase

AKR aldo-keto reductase

SDR short-chain dehydrogenase reductase

G6PDH glucose-6-phosphate dehydrogenase

HEK-293 human embryonic kidney 293 cell

PCR polymerase chain reaction

Q_RTPCR quantitative RealTime PCR

TLC thin layer chromatography

3α-diol 5α-androstane-3α, 17β-diol

4-dione androstenedione

ADT androsterone

DHEA dehydroepiandrosterone

DHEA-S dehydroepiandrosterone sulfate

El estrone

El-S estrone sulfate

E2 estradiol

T testosterone

## Authors' contributions

HL has participated in the design of the study and in drafting the manuscript; she has carried out the molecular biology manipulations, enzymatic assay, and the cell cultures. SFZ and GP carried out the *in situ *hybridization. VB carried out the partial enzymatic assays. FL has participated in careful reading of the manuscript. VLT conceived the study and was implicated in the redaction of the article. All authors read and approved final manuscript.
